# Delayed Diagnosis of Perrault Syndrome: A Rare Genetic Disorder

**DOI:** 10.1155/2024/5319443

**Published:** 2024-01-12

**Authors:** Mirgul Bayanova, Aigerim Abilova, Alisa Nauryzbayeva, Zhibek Turarbekova

**Affiliations:** “University Medical Center” Corporate Fund, Kerey, Zhanibek Khandar Str. 5/1, Astana, Kazakhstan

## Abstract

Perrault syndrome (PRLTS) is a rare autosomal recessive disorder which is associated with pathogenic variants in HSD17B4, HARS2, CLPP, LARS2, GGPS1, RMND1, TWNK, ERAL1, and PRORP genes. The disease is characterized by sensorineural hearing loss, sometimes with neurological signs, including progressive sensory and motor peripheral neuropathy, cerebellar ataxia, mild mental retardation, and ovarian dysgenesis in females. In this article, we report a case of a child diagnosed with spastic diplegic cerebral palsy. Determination of the segregation status of the parents of a proband with a rare compound heterozygote in the gene HSD17B4 will help the genetic counselling for the prognosis of Perrault syndrome in the family.

## 1. Introduction

M. Perrault described the first clinical report about two sisters with sensorineural hearing loss and infertility in 1951. Since then, Perrault syndrome has been recognized clinically and genetically as a heterogeneous disorder with an effect on gender, which has an autosomal recessive type of inheritance [1, 2]. There are two types: type I, which is static without neurologic disease, and type 2, which includes progressive neurologic illness [3]. The clinical manifestations are characterized by sensorineural hearing loss (SNHL) in both males and females and ovarian dysfunction in females. Sensorineural hearing loss is bilateral and ranges from profound with congenital onset to moderate with onset in early childhood. If the hearing loss begins in early childhood, it can be progressive. Ovarian dysfunction ranges from gonadal dysgenesis (absence or striated gonads) manifesting as primary amenorrhea to primary ovarian failure (POF) and resulting in early menopause. Fertility in affected men is reported to be expected (although the number of such cases is limited). In some cases, neurological symptoms develop among both men and women. Some examples are absent tendon reflexes, nystagmus, dysarthria, cognitive impairment, scoliosis, and cerebellar atrophy [4]. Recent reports of genes associated with Perrault syndrome have expanded the phenotypic manifestations to include pediatric metabolic disorders and leukoencephalopathy [5].

## 2. Case Presentation

**Figure 1:**
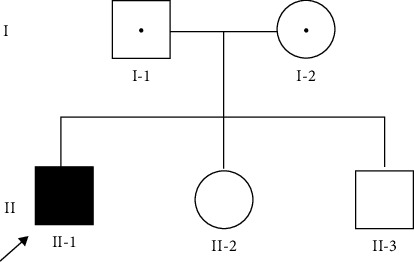
Pedigree of a family with Perrault syndrome. Proband (II-1), father (I-1), mother (I-2), and younger siblings (II-2, II-3).

A male proband of Asian ethnicity reported a complaint of regression of speech, delay in psycho-motor development, and inability to walk independently. According to medical records, the parents were 21 and 22 years old with no consanguinity at the time of conception, and it was the first pregnancy (Figure 1). Both parents were in good health and had no hearing problems. The pregnancy was uneventful, and the woman gave birth at 40 weeks (weight: 3500 grams and height: 53 cm). According to anamnesis from his mother, the child was growing and developing according to his age until the age of 2. By age 3, the child stopped walking and had difficulty in speaking. After seeing a neurologist, flaccid paraparesis was diagnosed. During four years, he was referred to UMC for further investigations and rehabilitation (National Research Center for Mothers and Child Health). It was recommended to have genetic testing by whole-exome sequencing (WES). Massive parallel sequencing revealed a compound heterozygous variant in HSD17B4, c.1333 + 2T > C, c.743G > A (Arg248His) (GenBank: NM_000414.4). We used Sanger sequencing by standard methodology [6] to validate for variants in the proband, identified by WES, and to verify the co-segregation variants in parents. Sanger sequencing was performed on a Genetic Analyzer 3500 (Applied Biosystems, Hitachi, Japan) with a BigDye Terminator v3.1 sequencing kit (Applied Biosystems, Foster City, CA, USA.

### 2.1. Investigations

Phenotype is as follows: stature is 110 cm, scoliosis, depressed nasal bridge, high palate, and bilateral sensorineural hearing impairment. Lower extremity muscle strength is reduced. Deep tendon reflexes examination is as follows: hyperreflexia in the arms and legs. Babinski's reflex is positive on both sides. Supported gait is paretic with flat walking of both feet. Presence of ankle joint stiffness.The patient does not understand instructions addressed to him or understands them partially during the examination. Speech is impaired, and vocabulary could be better. Mental development does not correspond to age.

The patient was subjected to radiological investigations. MRI shows signs of foci of gliosis in the frontoparietal lobes of the cerebral hemispheres (vascular, postischemic genesis probably) and retro cerebellar cyst. On electromyography (EMG) were found signs of distal minor axonopathy of the right deep peroneal nerve at the tarsal level with signs of focal demyelination. No evidence of proximal lesions on either side was obtained. Even so there was signs of mild distal demyelinating lesion of right tibial motor nervefibres. There is no evidence of femoral nerve involvement on either side. Notably, an audiologist had not seen the child before this age, and during the examination, it was found that the child had a grade 4 bilateral hearing loss (audiograms included).

To substantiate the clinical diagnosis, plasma levels of very long-chain fatty acids (VLCFA), di- and trihydroxycholestanoic acid (DHCA and THCA), and phytanic acid were measured. The result showed that all indicators were average, with 46 XY karyotypes.

**Figure 2:**
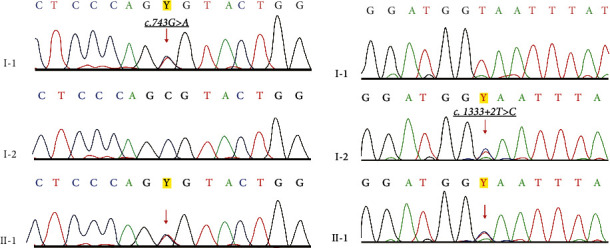
Sanger sequencing traces of family with Perrault syndrome. Parents (I-1, I-2) and proband (II-1).

Results of Sanger sequencing of 11 and 15 exons and exon-intron boundaries of the HSD17B4 gene revealed two potential sequence variants: c.1333 + 2T > C from his mother and c.743G > A from his father (Figure 2). The compound heterozygous mutation found confirms Perrault Syndrome 1 (OMIM: 233400). Variants were interpreted according to a 5-level classification system, recommended by the American College of Medical Genetics and the Association for Molecular Pathology (ACMG/AMP, on the Franklin platform (Franklin by Genoox, Genoox, USA)).

## 3. Discussion

The clinical heterogeneity of the disease makes diagnosis difficult because the HSD17B4 gene-encoded D-bifunctional protein (DBP) is involved in fatty acid b-oxidation and steroid metabolism, which can cause DBP deficiency (OMIM 261515) [7]. Patients with DBP deficiency present with hypotonia and seizures by one month of age, which is commonly fatal in early childhood. They have hearing and vision impairments and generally make no developmental progress [7]. In the case presented, a child with biallelic variants in HSD17B4 associated with a neurological phenotype and SNHL, we found no accumulation of b-oxidation substrates in serum, confirming our view of the diagnosis of PRLTS.

### 3.1. Outcome and Follow-Up

In our case, it is considered a golden catch as the patient came in with the main complaints of neurological symptoms. It was an oversight that his hearing has not been tested all these years. The child is currently being evaluated for hearing aids. The next step is to try the other children to find the same variants as probands and audiologist consultation. A pediatric endocrinologist and gynecologist consultation will be recommended for the proband's younger sister for exclusion of monadic dysgenesis by pelvic ultrasound until it is confirmed that she does not carry two variants.

### 3.2. Learning Points

- Patients with psycho-speech delay should additionally consult geneticists for timely referral to additional genetic tests and specialists.
- In heterogeneous manifestations of the disease, whole-exome sequencing allows a correct diagnosis. It is essential to know if it is a de novo or inherited mutation for the prognosis of offspring in the family.
- PRLTS is a clinically and genetically heterogeneous disease manifested by sensorineural hearing loss in both sexes and primary ovarian insufficiency in 46, XX karyotype females.

## References

[1] Faridi R., Rea A., Fenollar-Ferrer C. (2022). New insights into Perrault syndrome, a clinically and genetically heterogeneous disorder. *Human Genetics*.

[2] Domínguez-Ruiz M., García-Martínez A., Corral-Juan M. (2019). Perrault syndrome with neurological features in a compound heterozygote for two TWNK mutations: overlap of TWNK-related recessive disorders. *Journal of Translational Medicine*.

[3] Roberts L. M., Carnivale B. (2019). Perrault syndrome diagnosis in a patient presenting to her primary care provider with secondary amenorrhea. *Case Reports in Obstetrics and Gynecology*.

[4] Pan Z., Xu H., Tian Y. (2020). Perrault syndrome: clinical report and retrospective analysis. *Molecular genetics and Genomic Medicine*.

[5] Riley L. G., Rudinger-Thirion J., Frugier M. (2020). The expanding LARS2 phenotypic spectrum: HLASA, Perrault syndrome with leukodystrophy, and mitochondrial myopathy. *Human Mutation*.

[6] van Grunsven E. G., van Berkel E., Ijlst L. (1998). Peroxisomal D-hydroxyacyl-CoA dehydrogenase deficiency: resolution of the enzyme defect and its molecular basis in bifunctional protein deficiency. *Proceedings of the National Academy of Sciences of the U S A*.

[7] Chen S., Du L., Lei Y., Lin Y., Chen S., Liu Y. (2021). Two novel HSD17B4 heterozygous mutations in association with D-bifunctional protein deficiency: a case report and literature review. *Frontiers in Pediatrics*.

